# Some Recent Researches in the Diseases of the Domesticated Animals1Read before the twenty-seventh annual meeting of the United States Veterinary Medical Association, at Chicago, September 17th, 1890.

**Published:** 1890-10

**Authors:** D. E. Salmon


THE JOURNAL
OF
COMPARATIVE MEDICINE AND
VETERINARY ARCHIVES.

VOL. XI. OCTOBER, 1890. NO. 10.

### SOME RECENT RESEARCHES IN THE DISEASES OF THE DOMESTICATED ANIMALS.[1]

BY D. E. SALMON, D.V.M.

IT is not an easy thing to thoroughly investigate a germ disease, nor is it a task that can be accomplished, even with the best facilities, by a few weeks or a few months of work. It is fourteen years since Koch gave bacteriology a place among the sciences by his brilliant researches, which demonstrated the pathogenic relations of the *Bacillus anthracis* to the disease which we know as anthrax, and yet, in spite of the utmost activity in this field of work since that time, there are still many communicable diseases of the cause of which we are still in complete ignorance. To illustrate this, it is only necessary to mention the fact that the cause of such common diseases as smallpox and cowpox is still shrouded in mystery, notwithstanding the ease with which material for study can be obtained, and in spite of the large reward offered by the Grocers' Company for a successful method for artificially cultivating the vaccine virus.

Unfortunately, the greater part of the bacteriological researches which are published for the enlightenment of the world are absolutely valueless, and are a check to progress rather than an aid to it, because some one must disprove the conclusions which follow from them, and, even then, other workers must remain in doubt as to which observer was correct. The study of too small

[1] Read before the twenty-seventh annual meeting of the United States Veterinary Medical Association, at Chicago, September 17th, 1890.

a number of cases, the failure to use a sufficient number of experimental animals, the neglect of scientific methods, the lack of proper facilities for work, are among the most common causes of failure in bacteriological research. The most complete and thorough study of a single case of disease is not sufficient to enable any man to write a description of the typical symptoms and lesions of that malady. Individual cases in the same outbreak differ from each other to a surprising degree, while the type of one outbreak may differ very materially from that of another. These facts are well known, and for that reason the clinical history and pathological anatomy of any given disease cannot be considered reliable until many individual cases and a considerable number of epidemics or epizootics have been studied.

If this is true of the more superficial and easily observed characters, how much more is it to be remembered in connection with the obscure problems relating to the pathogenic germs, their morphology, physiology, life history, susceptibility to germicides, their effects upon the animal body in which they are multiplying, and the toxic products which are developed during their growth. These are questions which require long observation, a division of labor among competent assistants, many experiments, proper experimental grounds and laboratories, and every possible precaution to avoid errors and hasty generalizations. In speaking to you, therefore, of the recent bacteriological work of the Bureau of Animal Industry, I hope you will not anticipate any startling novelty which has been wrenched from nature's tenacious grasp within the last week or two, and which is destined to revolutionize or blot out all that we thought had been built up in the past. Such wonderful discoveries are few and far between, and when investigators claim them, the chances are that they are mistaken. My purpose is, rather, to point out results which we have reached by gradual and careful advances during years of patient work; and while such a plan on my part may debar me from introducing those sensational features which make much of our modern science resemble a fairy tale in more respects than one, I feel sure that there are some advantages in dealing with conclusions which are reasonably well founded upon carefully observed facts.

Realizing the impossibility of thorough work being done by any one man in so difficult and complicated a field, it has been my constant endeavor to divide the investigations, so that each branch may be in the hands of a specialist. Thus, it is the duty of one veterinarian in the bureau to secure proper animals for experiment, to take charge of such animals during the experiments, take their temperature, observe their symptoms, and see that there is no chance of contagion from lot to lot. Another man, with from three to five assistants, has general charge of the investigations, makes the cultures, prepares the material for inoculation, studies the germs and the pathology of the disease, and decides what experiments are necessary to elucidate these points. The chemical investigations are carried out by a competent chemist, and an artist is always ready to make illustrations of the pathological appearances or of the germs, while still another person is prepared to photograph such specimens as must be delineated with absolute correctness. In addition to this, we have men to collect material and observe outbreaks in the field, so that our experimental work will not suffer by constant interruption. If one man undertakes to carry on all of these duties, his time is broken up, his experimental animals are neglected and his experiments themselves interrupted. This accounts for much of the poor work that is being done, and shows the necessity of at least one place in the country where investigations are carried on with every facility in the way of experimental grounds, laboratories, men and money to give reliable results.

In working for the development of such an institution as this at Washington, my chief object has been to show what services the veterinary profession can render to the country and to the world, and in doing this to incidentally furnish the means for protecting our animals from disease, and the facts for a more substantial, complete and scientific foundation for veterinary practice. In carrying out this plan I have had the cordial coöperation and assistance of the profession, and I take the opportunity to return thanks for this unfaltering support, and to express the hope that our labors may be so performed as to merit a continuance of a like good-will in the future.

The chief part of our scientific work has been for so long a time devoted to swine diseases that it is impossible to omit a consideration of them in a paper of this kind. You are aware that we have described two diseases caused by two distinct germs. These germs can be easily distinguished from each other, but we are now satisfied that the diseases can only be safely diagnosed by determining which germ is present. Different outbreaks of swine plague differ greatly in the lesions presented. Sometimes these are confined to the lungs, as in the first cases we studied; often, however, the intestines are affected, and the appearances presented may then so closely resemble those seen in hog cholera that the most practiced eye cannot decide which germ is responsible for the mischief. It is then only by a bacteriological examination that an accurate diagnosis can be made.

These diseases—though frequently both are present in the same herd—are often found each by itself producing ravages without the aid of the other. In a very destructive outbreak studied during the present Summer, in New Jersey, the most careful bacteriological examination revealed only the swine-plague germ. Some of the animals from this outbreak were taken to our experiment station, and placed in an enclosure with other pigs, and the disease was communicated to the latter by cohabitation. There is, consequently, no doubt about swine plague being a communicable disease—one that can be transmitted by inoculation and by cohabitation.

Its relative distribution and prevalence as compared with hog cholera are questions that remain to be answered. If the lesions were always so characteristic that a diagnosis could be made by an ordinary *post-mortem* examination, it would be an easy thing to put a force of men in the field and determine them in a single season, but so long as a bacteriological study must be made of each case it, of course, requires a much longer time to obtain satisfactory results. My first impression was that swine-plague was of much less importance from an economical point of view than hog cholera, but our recent investigations have modified these views, and have indicated that the former disease may be as prevalent and cause as heavy losses as the latter.

The great aim in bacteriological investigation, as in all other branches of medical science, is to discover the means of preventing or curing diseases. The discovery of the microbe and the study of its life history are but steps in the accomplishment of this object. We have, therefore, from the first, endeavored to bring out such facts as would enable us to form a science for the prevention of germ diseases.

It was once my good fortune to listen to a series of lectures by that great naturalist, Louis Agassiz, and I was deeply impressed with a statement of his to the effect that Nature held her secrets with so tight a grasp that an investigator might consider his life successful if, during the whole course of it, he had been able to discover and contribute to science but a single new principle. He himself, after a life of almost unparalleled activity in scientific research, laid stress upon but one such discovery, although he had contributed an enormous mass of facts. In our investigations we have discovered two such principles, which have been and are destined to be of very great value to sanitary science.

The first of these principles is that with nearly all diseases the most susceptible animals have a certain power of resisting germs of the greatest virulence. Or, putting the same idea in other words, we may say that by diminishing the dose of virus we reach a point where a considerable number of germs may be introduced into the tissues without being able to cause disease. The second great principle is that with non-recurrent diseases the germs produce during their cultivation outside of the animal body certain chemical substances which, when administered to susceptible animals, grant immunity from the effects of that germ. These principles, discovered and demonstrated in the course of the investigations made under our Department of Agriculture, promise to be of more value to sanitary science than all the other principles which the combined workers of the world have contributed to bacteriology since Koch demonstrated the germ theory of disease.

The easy, safe and certain production of immunity in animals exposed to contagion would be a most powerful weapon for the control of germ diseases, and, consequently, our investigations have been for years turned in this direction. We have tried the Pasteur method of vaccination, using attenuated virus or vaccine ; we have tried inoculation on the principle discovered by us of producing the desired effect by means of graduated doses of strong virus, and we are now experimenting with the chemical substances produced during the growth of the microbes. We have found that vaccination and inoculation, as applied in hog cholera, are uncertain and insufficient in results and have certain disadvantages which make these methods of little value for the prevention of this disease. We turn, therefore, to the method of prevention by means of the chemical substances formed during bacterial multiplication.

This question is one of the most difficult ones to study that has confronted the bacteriologist. When we first demonstrated the principle we found that the culture liquid when freed from living germs had the power of conferring immunity when injected into the tissues. But this very discovery suggested a host of questions which must be answered before the method could be of practical use. The culture liquid, considered chemically, is a complex substance. Which of its constituents has this wonderful power? What are its properties? How can it be separated from the useless matter with which it is combined? Is it a single alkaloidal substance, or does the result depend upon two or more of these combined? At what period of bacterial growth is it most abundant? Will it produce the desired effect when administered in other ways than by hypodermic injection? What is the dose required to produce satisfactory immunity in the hog, and how often and at what intervals must this dose be repeated? These are some of the most important questions which pressed themselves upon us for solution; and when we attempted to solve them we found other questions of a secondary interest which we were compelled to grapple with and solve before the others could be undertaken.

We had no test for this mysterious and hidden substance, no way to recognize its presence except its effect in the production of immunity, and this test it required weeks to make, and even then the experiments were liable to fail. How much more patience and skill does such an investigation require than the ordinary chemical methods where a reagent is added to a solution and the immediately formed precipitate shows the presence of the sought-for constituent! Our first experiments were made with pigeons, and while they served to demonstrate the principle, we soon found, to use chemical language, that the reaction with them was not a sufficiently delicate one to enable us to solve, without great loss of time, the other questions that came before us. The difficulty was that pigeons are not easily killed by the hog cholera germ, especially in Summer, and, consequently, after we had treated some to produce immunity and afterward inoculated them together with other pigeons to test their comparative resistance, none of the inoculated birds would die, the untreated ones resisting as well as those that had received treatment. Such an experiment was, of course, a failure and must be repeated.

In short, it was necessary for us to select an animal to experiment with that in its natural condition of resistance would always die when inoculated with a moderate dose of virus, but which, with a slight addition to its natural power of resistance, would always recover from such an inoculation. The pig was out of the question—its resistance to virus is too variable and the possibility of producing immunity too doubtful. After considerable experimentation, we found the guinea-pig to answer these conditions to our satisfaction. It was here that our first discovered principle came to our assistance. We so graduated the dose of strong virus used in our tests that we gave just sufficient to produce death in the natural condition of resistance. Our guinea-pigs might then be compared to the chemist's reagent, which is just faintly alkaline. He dips in it the litmus paper and it remains blue; but let him add but a single drop of acid and then touch it with the litmus paper, and the red color at once reveals the acidity. So with the guinea-pigs—inoculate them with our standard dose of virus and they invariably die; but add only slightly to their power of resistance and all will live. I cannot go into full details of our experiments in this paper; in fact, they are still in progress, but I have already given you an idea of our greatest difficulty, and how we overcame it.

Another difficulty was to get the material for producing immunity in a sufficiently concentrated form so that a large enough dose could be given hypodermically. The culture liquid contains such a small proportion of it, and the dose of liquid injected must, consequently, be so large, that it could not well be given to small animals hypodermically. We tried first to inject it in sufficient quantity in the abdominal cavity, but it is so irritating in its properties as to frequently produce peritonitis and death. We also tried to condense the liquid by evaporation, but our substance was either volatile or destroyed by the heat, for our condensed liquids did not show a corresponding increase in the power of conferring immunity. At this stage of the investigations we added a chemist to our force, and have since learned not only how to condense our culture liquids without destroying their properties, but we have separated the chemical constituents and decided which are concerned in the production of immunity. There are many details which must still be worked out before the method can be practically applied to swine, or before we know that hog cholera can be practically prevented by this means. The most difficult questions of the investigation, however, are solved, and what remains is a matter of detail. These statements are made because this is the most interesting line of bacteriological work now engaging the attention of scientists, and I know that you will be interested in learning something of the great problems before us and how we have solved them.

We will now turn our attention for a few minutes to the subject of Texas fever. While this disease, as you know, is allied by its characters to the bacterial fevers, it has certain marked peculiarities which have caused it to be regarded as the most mysterious malady which remains for modern science to investigate. For four years we have been giving the disease very close attention and study, and our results have been unexpected to ourselves, and so different from those reached by other investigators, that we have not pressed our conclusions until we had taken every precaution to confirm them.

I am prepared now to say that Texas fever is not a bacterial disease. There are no peculiar bacteria to be found in the blood, spleen, liver, or other organs; and as a rule the blood and tissues are free from even the common septic bacteria when examined immediately after the death of the affected animals. All the illustrations which have been published showing preparations of blood from Texas fever animals swarming with bacteria, and sections of the tissues showing the same micro-organisms, are misleading, and may be put aside as of no value to the student of this disease. The blood and tissues do not present such appearances when properly examined.

Texas fever belongs to the malarial type of diseases. The germ resembles the malarial organisms of Laveran. It is found within the red corpuscles of the blood, and may be demonstrated in them before the death of the animal. We have not been able to cultivate it, and do not believe that it can be cultivated by any of the methods now in use by bacteriologists.

We have studied outbreaks that were caused by cattle from the tide-water section of Virginia, from North Carolina and from Texas, and in every case we found the same micro-organism present in the blood. In some cases almost every corpuscle would be invaded. These parasites rapidly destroy the corpuscles, and the number of these soon falls to one-half, one-third, one-fourth or even one-fith of what is found in health. This destruction of the corpuscles, of course, accounts for the excretion of the red coloring matter in the urine.

The possibility of the infection of pastures by ticks has also been demonstrated. For two years we have picked the ticks from Southern cattle and scattered them over experimental pastures and we have found that susceptible cattle would contract the fever from these pastures as readily as from those upon which the Southern cattle themselves had been placed. It is a fact, therefore, that ticks may infect pastures without the presence of Southern cattle. Whether Southern cattle can infect pastures without the presence of ticks is still an open question. We had an experiment planned to settle this problem the present Summer, but it failed by the unexpected appearance of the ticks upon this lot of cattle which we had supposed were protected from them.

The main results of our Texas fever investigations may therefore be summed up as follows:

Texas fever belongs to the malarial group of diseases.

It is caused by small micro-organisms which are found in the red blood-corpuscles, and which are not bacteria, but bodies of the nature of the malarial germs of Laveran.

Pastures may be infected by ticks from the bodies of Southern cattle.

While this is only a beginning of the work necessary to elucidate the many interesting and important questions connected with Texas fever, it gives us a solid foundation from which to build, and indicates the direction in which future researches must be made in order to secure successful results.

